# Clinical Outcomes of Salvage Endoscopic Nasopharyngectomy for Patients With Advanced Recurrent Nasopharyngeal Carcinoma

**DOI:** 10.3389/fonc.2021.716729

**Published:** 2021-07-22

**Authors:** Wanpeng Li, Huankang Zhang, Hanyu Lu, Huan Wang, Yurong Gu, Houyong Li, Xicai Sun, Hongmeng Yu, Dehui Wang

**Affiliations:** Department of Otolaryngology-Head and Neck Surgery, Affiliated Eye Ear Nose and Throat Hospital, Fudan University, Shanghai, China

**Keywords:** recurrent nasopharyngeal carcinoma, endoscopic, nasopharyngectomy, survival, prognostic factors

## Abstract

**Background:**

Salvage endoscopic nasopharyngectomy has better survival prognosis and fewer complications in the management of early stage rNPC, compared to re-irradiation. However, the treatment modality of advanced recurrent nasopharyngeal carcinoma (rNPC) remains controversial. Thus, the purpose of this study was to investigate the demographics, clinical outcomes, and prognostic factors associated with salvage endoscopic nasopharyngectomy in advanced rNPC.

**Methods:**

This study conducted a retrospective analysis of advanced rNPC patients who underwent salvage surgery betweenm January 2014 and December 2019. The overall survival (OS) and progression-free survival (PFS) were analyzed. Univariable and multivariable analyses of OS and PFS were performed using the Cox regression model. The predicted values of the parameters were determined by means of the receiver operating characteristic (ROC) curve analysis.

**Results:**

Among the 120 patients included, there were 75 patients with rT3 stage and 45 patients with rT4 stage. With the median follow-up time of 18 months,the 3 -year OS and PFS were 55.2% and 29.4%, respectively. Multivariate analyses showed that the rNPC patients with older age, low BMI (Body Mass Index), rT4 stage, tumor necrosis, and tumor invasion into the ICA was predictive of worse OS, whereas low BMI and rT4 stage were associated with worse PFS. In addition, the rT stage was identified as a better predictor of OS (area under the ROC curve: 0.669; *P*=0.003) than the other clinical features.

**Conclusions:**

Salvage treatment using endoscopic nasopharyngectomy appears to be an effective treatment in the management of patients with advanced rNPC. In addition, case matching studies and prospective studies with larger clinical samples are required to further evaluate the efficacy of endoscopic surgery compared with re-irradiation in advanced rNPC.

## Introduction

Nasopharyngeal carcinoma (NPC), an epithelial malignant tumor originating from the mucosal lining of the nasopharynx, is endemic in Southern China and Southeast Asia (1). As a result of the advancements in the field of intensity-modulated radiotherapy (IMRT) in the management of patients with NPC, local recurrence remains one of the most important modes of treatment failure. Overall, 10% to 15% of the patients who undergo definitive radiotherapy will display local recurrence after undergoing treatment (2). Re-irradiation with IMRT for the management of recurrent nasopharyngeal carcinoma (rNPC) is often accompanied by serious complications, such as radiation necrosis of the bone, multiple cranial nerve dysfunction, and brain necrosis, which damages the quality of life of the patients and even leads to death (3). Moreover, Yu et al. reported that treatment with re-irradiation for the management of rNPC could only improve the survival rates in the patients with rT1-2 disease, whereas no significant changes were observed in the patients with rT3-4 disease (4).

Several recent studies have investigated the employment of salvage endoscopic nasopharyngectomy in the management of rNPC, in view of the fact that it might have better survival prognosis and fewer complications, compared to re-irradiation (5, 6). Liu et al. reported that endoscopic surgery significantly improved the overall survival (OS), compared to IMRT, in the patients with early stage rNPC (tumors confined to the nasopharyngeal cavity, the postnaris or nasal septum, the superficial parapharyngeal space, or the base of the sphenoid sinus) (7). Furthermore, a previous study by the authors on patients with rNPC showed that the site-specific and sinonasal-related quality of life were maintained after endoscopic nasopharyngectomy, compared to the preoperative quality of life. Endoscopic surgery appears to be a valuable therapeutic option in the management of patients with rNPC (8).

In scenarios involving advanced rNPC (rT3 and rT4 stages), endoscopic surgery is a challenging endeavor, owing to the frequent invasion into the internal carotid artery (ICA), skull base, orbit, infratemporal fossa, dura mater, cranial nerves, etc. Previous literature reports on the subject are few and involved small sample sizes. Consequently, the survival prognosis and the independent prognostic factors remain controversial (9, 10). The current study retrospectively collected the data pertaining to 120 patients with advanced rNPC who underwent endoscopic nasopharyngectomy during the time period from January 2014 to December 2019. To the best of our knowledge, the present study involves the highest number of reported cases of endoscopic surgery for the management of advanced rNPC. The present study focused on the demographics, clinical outcomes, and prognostic factors pertaining to these patients with rNPC.

## Methods

### Study Population

The present study performed a retrospective chart review of 120 patients who were diagnosed with advanced rNPC and underwent endoscopic nasopharyngectomy for the management of the same at the Department of Otorhinolaryngology of the Affiliated Eye, Ear, Nose, and Throat Hospital at Fudan University during the time period from January 2014 to December 2019. The patients with T1 and T2 rNPC, and missing data pertaining to important variables were excluded from the study. The reasons for the missing data pertaining to important variables included incomplete preoperative clinical data and inability to contact the patient (e.g., phone number was no longer valid). In addition, there are also some cases without surgically resectable for patients with advanced rNPC: (1) The tumors extensively invaded the intracranial structure, especially the important blood vessels and nerves; (2) The tumors invaded the intracranial structure with obvious radiation brain edema after previous radiotherapy; (3) distant metastasis; (4) unresectable neck lymph node metastasis. The tumor margin to the ICA of less than 0.5 cm was considered as tumor invasion into the ICA (7). The current study was approved by the Institutional Review Board of the Affiliated Eye, Ear, Nose, and Throat Hospital at Fudan University.

### Salvage Endoscopic Nasopharyngectomy

The decision to perform salvage endoscopic nasopharyngectomy was based on the location and extent of the tumor, taking into account the patient preferences and consultations with the radiation oncologists and surgeons. In case of the lesions involving the sphenoid sinus, bilateral sphenoidal sinuses were opened, and the septum was removed. The posterior end of the nasal septum was removed, and the base of the sphenoid sinus was ground off to outline the sphenoid sinus and nasopharynx. In order to remove the tumors invading the base of the middle cranial fossa and infratemporal fossa, a modified Caldwell-Luc approach through the anterior wall of the ipsilateral maxillary sinus into the infratemporal fossa and the base of the middle cranial fossa was often required to create a better surgical space and to complete the surgical procedure. The external pterygoid plate was abraded, and the foramen ovale and the main trunk of the mandibular nerve were exposed backwards. Bleeding from the pterygoid venous plexus was arrested by packing with quick yarn. The lingual and inferior alveolar nerves on the posteromedial side of the external pterygoid muscle were located, and the middle meningeal artery and sphenoid spine were exposed posteriorly.

The skull base bone can be divided into cancellous substance and cortex. When the tumor invades the cancellous bone of skull base, sometimes it is not easy to distinguish from the inflammatory granulation tissue after radiation. Pathological biopsy can be taken during the operation to determine whether there is tumor invasion, so as to ensure the negative margin. When the tumor invades the cortex of the skull base, surgeons can judge the extent of tumor invasion by endoscopic visual field and grind the invaded bone to the normal boundary by drill.

**Figure 1:**
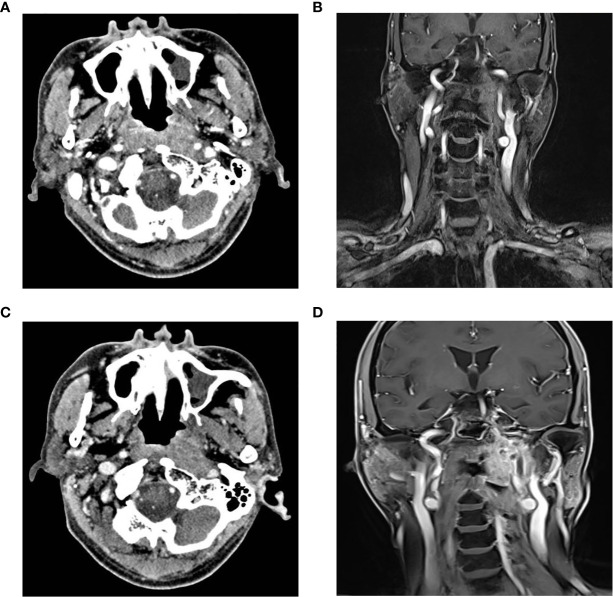
Imaging examination of ICA before and after embolization **(A)** Horizontal enhanced CT showed that the tumor partially wrapped the left ICA; **(B)** Coronal enhanced MRI exhibited that the tumor invaded the left pharyngeal recess, parapharyngeal space, the longus capitis, sphenoid body and skull base, and partially surrounded the left ICA; **(C, D)** Horizontal enhanced CT and coronal enhanced MRI showed the undeveloped cavenous, lacerum, petrous and cervical segments of the left ICA. CT, Computer Tomography; MRI, Magnetic resonance imaging; ICA, Internal carotid artery.

**Figure 2:**
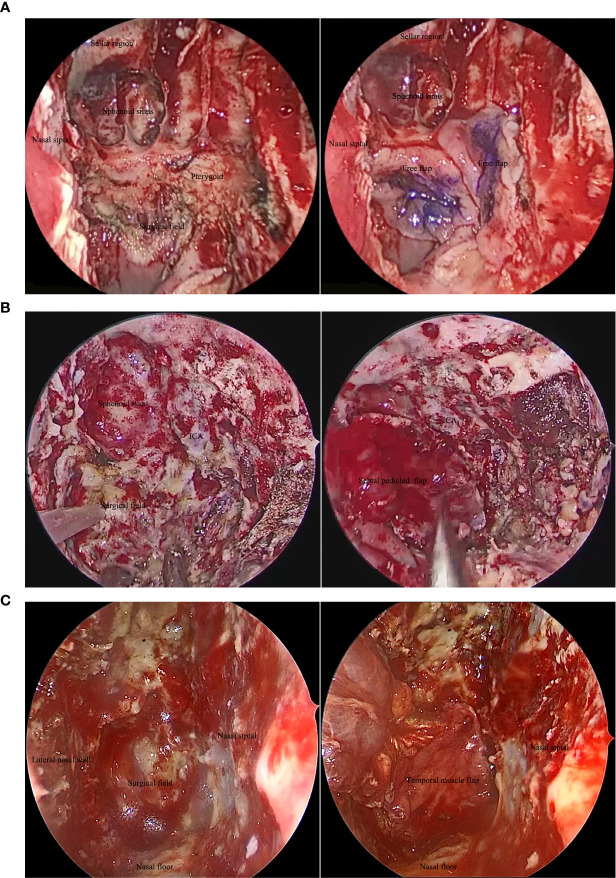
Reconstruction of skull base defect after salvage surgery for recurrent nasopharyngeal carcinoma: **(A)** nasal free flap; **(B)** septal pedicled flap; and **(C)** temporal muscle flap. ICA, Internal carotid artery.

In cases with tumor invasion into the internal carotid artery (ICA), some patients underwent balloon occlusion test (BOT) of the ICA prior to the surgical procedure. If BOT was negative, ICA occlusion could be performed immediately, and the lesion could be removed during surgery (**Figure 1**). The parapharyngeal, petrosal, foramen, and clival segments of the ICA were exposed, and the related ICA was resected, in accordance with the extent of invasion. If BOT was positive, bypass surgery between the external carotid artery and middle cerebral artery was performed prior to the tumor resection. Subsequently, the rNPC resection was performed after two weeks. In order to prevent postoperative complications, such as wound infection caused by the extensive exposure of the skull base, nasal free mucosal flap, septal pedicled mucosal flap, or temporal muscle flap were used to repair the skull base defect (**Figure 2**).

### Clinical Data

The current study retrieved the following clinical data pertaining to the patients: the demographic and clinical features such as the age, sex, history of smoking, alcohol consumption, diabetes, hypertension, and body mass index (BMI); tumor-related features including the number of radiotherapy sessions before surgery, preoperative combined chemotherapy, the time interval between recurrence and the last session of radiotherapy, rT stage, status of lymph node metastasis (LNM) at the time of recurrence, pathological type, status of tumor necrosis, surgical margins, tumor invasion into the ICA, the use of pedicled flaps to repair the skull base defect, postoperative adjuvant therapy, progression-free survival (PFS) and overall survival (OS); and serological parameters, i.e., hemoglobin (Hb) levels, neutrophil-to-lymphocyte ratio (NLR), and serum alkaline phosphatase levels.

### Statistical Analysis

The curves of OS and PFS were plotted using the Kaplan–Meier method, and the significance of the differences among rNPC patients with regard to the prognostic factors was analyzed using the Log-rank tests. A Cox regression model was used to perform multivariate survival analyses. The predicted values of the parameters were determined by means of the receiver operating characteristic (ROC) curve analysis. The follow-up period was defined as the time period from the initial diagnosis at our institution to the date of death or last contact.

## Results

### Study Population

A summary of the details regarding the patients involved in the present study is presented in **Table 1**. A total of 120 patients were identified; 88 (73.3%) male subjects and 32 (26.7%) female subjects. Among the aforementioned patients, the number of patients with age ≥50 years, a history of smoking, alcohol consumption, diabetes, and hypertension were sixty-five, thirty, twenty-one, four, and thirty, respectively. In the present study, the normal range of BMI was set at 18.5–24.9 kg/m^2^; 26, 80, and 14 patients had high, normal, and low BMI, respectively. Regarding the serologic parameters, forty-two, forty, and six patients had low hemoglobin (<120 g/L) levels, high NLR (≥6), and low serum alkaline phosphatase (<50 mmol/L) levels, respectively. The time interval between recurrence and the last radiotherapy session was more than three years in sixty-eight patients.

**Table 1:** Characteristics of patients with advanced rNPC.

Characteristics	Total = 120	%
Gender		
Male	88	73.3
Female	32	26.7
Age (years)		
≥50	65	54.2
<50	55	18.3
Smoking history		
Yes	30	25.0
NO	90	75.0
Drinking history		
Yes	21	17.5
No	99	82.5
Diabetes mellitus		
Yes	116	96.7
No	4	3.3
Hypertension		
Yes	90	75.0
No	30	25.0
BMI		
18.5–24.9	80	66.7
<18.5	14	11.7
>24.9	26	21.7
Hemoglobin		
≥120	78	65.0
<120	42	35.0
NLR		
≥6	40	33.3
<6	80	66.7
Alkaline phosphatase		
<50	6	5.0
≥50	114	95.0
Period between recurrence and the last session of radiotherapy (year)		
≥3	68	56.7
<3	52	43.3
Preoperative combined chemotherapy		
Yes	99	82.5
No	21	17.5
Histological subtype		
WHO type II	43	35.8
WHO type III	77	64.2
rT stage		
rT3	75	62.5
rT4	45	37.5
LNM		
Yes	28	23.3
NO	92	76.7
Tumor necrosis		
Yes	64	53.3
No	56	46.7
Tumor invasion of the ICA		
No invasion	79	65.8
Invasion	28	23.3
Invasion, but ICA was embolized	13	10.8
The use of pedicled flap		
Yes	55	45.8
No	65	54.2
Surgical margin		
Negative margin	85	70.8
Positive margin	35	29.2
Postoperative radiotherapy		
No	112	93.3
Yes	8	6.7
Postoperative chemotherapy		
No	94	78.3
Yes	26	21.7
Postoperative PD-1 treatment		
No	111	92.5
Yes	9	7.5
All patients with follow-up (mean months of survival) range)	18 (2-81)	
Outcome		
Remission	39	32.5
Deceased	40	33.3
Alive with disease	41	34.2

rNPC, recurrent nasopharyngeal carcinoma; BMI, Body mass index; NLR, Neutrophil to lymphocyte ratio; LNM, lymph node metastasis; ICA, Internal carotid artery.

Among the study subjects, 99 patients received adjuvant chemotherapy along with preoperative radiotherapy. The most frequent histological subtype of the tumor encountered in the current study was World Health Organization (WHO) type III (n=77, 64.2.0%), followed by WHO type II (n=43, 45.8%). In the current study, the tumors were staged on the basis of the rTNM staging system by the American Joint Committee on Cancer (AJCC/UICC) (7th edition, published in 2010) as follows: rT3 (n=75) and rT4 (n=45). The current study detected lymph node metastases in 28 patients (23.3%). The presence of tumor necrosis was observed in 64 patients (53.5%).

Preoperative tumor invasion into the ICA was observed in 41 patients (34.2%), including 13 cases with ICA embolization and 28 cases without ICA embolization. Moreover, in 79 patients, the tumor did not invade the ICA (63.8%). Among the 120 patients, 84 (70.0%) underwent repair of the postoperative nasopharyngeal defect. The pedicled flap was used in 55 cases (45.8%), including 41 cases of pedicled septal mucosal flap and 14 cases of temporal muscle flap. The pedicled flap was not employed to repair skull base defect in 65 patients, including 29 cases that underwent repair using nasal free mucosal flap and 36 cases without any intervention. Furthermore, eight, twenty-six, and nine patients received postoperative radiotherapy, chemotherapy, and programmed cell death protein 1 (PD-1) treatment, respectively.

Among the study subjects, 85 (70.8%) patients had negative surgical margins. The relationship between surgical margin and ICA embolization in patients with tumor invasion of the ICA is shown in **Table 2**. A higher rate of negative margins (11/13, 84.6%) was observed in the patients with ICA embolization, compared to those without embolization (15/28, 53.6%). However, there was no significant difference between the two groups (P=0.084).

**Table 2:** The relationship between surgical margin and ICA embolization in rNPC patients with tumor invasion of ICA.

Embolization of ICA	Surgical margin		P value
	Negative margin	Positive margin	
No (n)	15	13	0.084
Yes (n)	11	2

rNPC, recurrent nasopharyngeal carcinoma; ICA, Internal carotid artery.

### Postoperative Complications

In this study, no patient died during the perioperative period. Although the post-operative period was uneventful, there still remain some complications. Most patients have wound scab, dry nose and headache in the first few days after surgery. The use of antibiotics and nasal irrigation gradually relieved these symptoms in about 4 weeks. In addition, 16 cases (13.3%) developed nasopharyngeal necrosis, which is a serious postoperative complication caused by surgical wound infection and difficult to heal due to previous radiotherapy. 15 patients (12.5%) had dyskinesia of masticatory muscle or limitation of mouth opening due to the mandibular branch injury of trigeminal nerve. One patient occurred cerebral infarction after ICA embolization.

### Overall Survival and Progression-Free Survival

**Figure 3:**
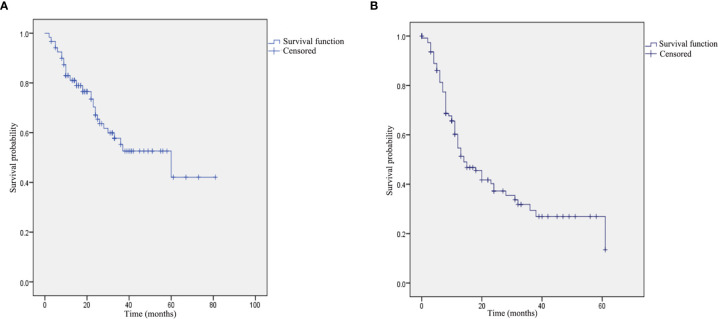
Kaplan–Meier curve pertaining to the survival in patients with advanced recurrent nasopharyngeal carcinoma: **(A)** overall survival: and **(B)** progression-free survival.

The median duration of follow-up in the current study was 18 months (range: 2–81 months). Among the 40 patients who expired during the course of the study, 19 patients expired due to tumor progression at the local site (n=12) or the brain (n=5), the cause of mortality in 15 patients was postoperative ICA hemorrhage, three patients expired due to lung metastasis, one patient died as a result of skull base necrosis, one patient died of brain necrosis, and one patient expired by reason of cerebral infarction. Remission was confirmed by means of physical examination and enhanced magnetic resonance in 39 patients (32.5%), and 41 patients (34.2%) survived with the disease. The 1-year,2-year and 3-year OS pertaining to all the patients were 81.1%, 67.1% and 55.2%, respectively. In addition, the 1-year, 2-year and 3-year PFS were 54.7%, 37.3% and 29.4%, respectively (**Figure 3**).

### Prognostic Analysis

**Figure 4:**
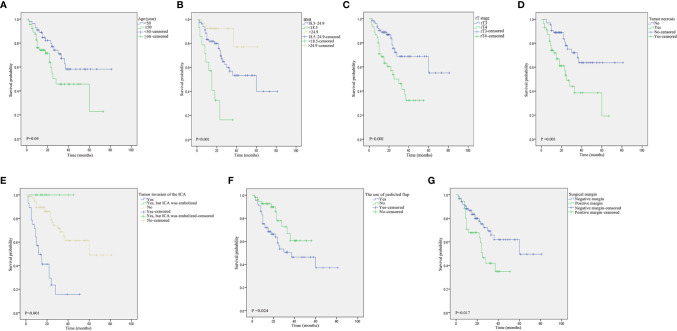
Kaplan–Meier curves pertaining to the overall survival in patients with recurrent nasopharyngeal carcinoma: **(A)** age (≥50 *vs*. <50 years); **(B)** body mass index (BMI); **(C)** rT stage (rT3 *vs*. rT4); **(D)** tumor necrosis; **(E)** tumor invasion into the ICA; **(F)** the use of pedicled flap; and **(G)** surgical margins.

**Figure 5:**
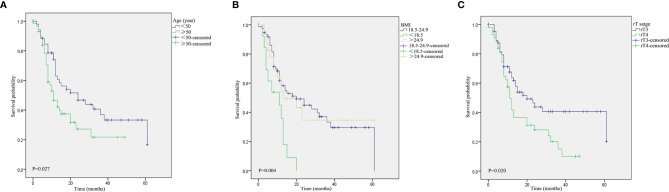
Kaplan–Meier curves pertaining to the progression-free survival in patients with recurrent nasopharyngeal carcinoma: **(A)** age (≥50 *vs*. <50 years); **(B)** body mass index (BMI); and **(C)** rT stage (rT3 *vs*. rT4).

The log-rank tests for prognostic factors pertaining to advanced rNPC are shown in **Table 3**. Age ≥50 years (**Figure 4A**), low BMI (**Figure 4B**), rT4 disease (**Figure 4C**), tumor necrosis (**Figure 4D**), tumor invasion into the ICA (**Figure 4E**), the use of pedicled flap (**Figure 4F**) and positive surgical margins (**Figure 4G**) were associated with a poor prognosis of OS. In addition, Age ≥50 years (**Figure 5A**), low BMI (**Figure 5B**) and rT4 disease (**Figure 5C**) were found to be significantly associated with worse PFS.

**Table 3:** Log-rank test of prognostic factors for rNPC.

Prognostic factors	OS (%)			P value	PFS (%)			P value
	1-year	2-year	3-year		1-year	2-year	3-year	
Sex				0.110				0.640
Male	83.5	73.8	60.4		56.9	36.0	31.4	
Female	74.1	61.4	45.5		48.1	39.2	23.5	
Age (year)				0.040				0.027
≥50	74.1	54.9	45.7		46.6	27.2	21.7	
<50	88.9	77.1	62.7		63.0	46.7	37.0	
Smoking history				0.120				0.127
Yes	89.3	80.3	70.3		55.6	41.1	33.2	
NO	78.2	63.4	51.8		51.4	24.0	18.0	
Drinking history				0.220				0.846
Yes	90.5	90.5	77.6		54.1	37.1	29.8	
NO	79.1	63.2	51.9		57.1	38.1	28.6	
Diabetes mellitus				0.926				0.815
Yes	100	50	50		66.7	33.3	33.3	
NO	80.5	67.3	55.4		54.0	37.2	29.3	
Hypertension				0.847				0.670
Yes	82.9	78.0	59.4		54.6	31.9	31.9	
No	80.4	63.9	54.4		54.6	38.8	28.9	
BMI				<0.001				0.004
18.5–24.9	81.7	69.5	53.2		58.2	44.9	33.3	
<18.5	57.1	32.7	16.3		35.9	0	0	
>24.9	92.3	92.3	92.3		54.4	34.6	34.6	
Hemoglobin				0.461				0.115
≥120	81.5	67.9	57.5		59.4	41.2	33.7	
<120	80.2	64.9	47.3		45.9	32.2	16.1	
NLR				0.137				0.644
≥6	72.5	56.1	42.1		38.7	38.7	29.0	
<6	83.4	70.2	58.7		59.0	36.7	29.6	
Alkaline phosphatase				0.899				0.428
<50	83.3	83.3	41.7		60.0	60.0	60.0	
≥50	81.0	66.2	55.6		54.3	36.1	28.0	
Preoperative combined chemotherapy				0.067				
chemotherapy								0.541
Yes	84.1	69.2	57.4		52.4	40.7	31.6	
No	66.7	55.6	41.7		65.9	13.8	13.8	
Period between recurrence and the last								
session of radiotherapy (year)				0.433				0.291
≥3	80.4	72.4	68.8		60.6	43.7	30.9	
<3	81.8	62.9	43.2		47.9	30.6	27.2	
Histological subtype				0.406				0.340
WHO type II	73.9	63.5	57.7		59.0	44.1	35.3	
WHO type III	86.6	69.2	54.5		52.2	34.1	26.0	
rT stage				0.001				0.020
rT3	88.8	75.3	68.8		63.7	43.8	40.6	
rT4	68.4	53.7	36.9		41.8	28.2	15.1	
LNM				0.216				0.159
Yes	78.2	60.0	40.0		55.7	28.6	14.3	
No	81.9	69.2	60.0		54.1	40.4	33.1	
Tumor necrosis				0.001				0.698
Yes	71.8	53.4	38.6		58.7	36.2	25.6	
No	88.8	78.0	67.9		50.4	39.6	39.6	
Tumor invasion of the ICA				<0.001				0.192
No invasion	89.6	79.2	65.1		57.6	39.6	34.8	
Invasion	49.5	23.6	15.7		39.9	23.3	11.7	
Invasion, but ICA was embolized	100	100	100		68.6	57.1	28.6	
The use of pedicled flap				0.024				0.513
Yes	92.6	77.8	60.5		50.9	33.7	23.6	
No	71.8	58.7	50.5		57.6	40.2	34.0	
Surgical margin				0.017				0.139
Negative margin	86.6	75.2	61.9		55.2	43.0	36.3	
Positive margin	67.8	50.8	41.9		53.4	25.1	15.1	
Postoperative radiotherapy				0.655				0.200
No	81.5	68.3	55.6		56.6	38.1	29.6	
Yes	75.0	50.0	50.0		31.3	31.3	31.3	
Postoperative chemotherapy				0.134				0.248
No	79.1	65.8	51.1		58.3	39.1	34.0	
Yes	87.7	71.7	71.7		42.4	31.4	16.7	
Postoperative PD-1 treatment				0.238				0.675
No	79.5	66.2	53.7		54.9	38.3	32.5	
Yes	100	83.3	83.3		51.9	25.9	25.9	

rNPC, recurrent nasopharyngeal carcinoma; OS, Overall survival; PFS, Progress free survival; BMI, Body mass index; NLR, Neutrophil to lymphocyte ratio; LNM, lymph node metastasis; ICA, Internal carotid artery.

**Figure 6:**
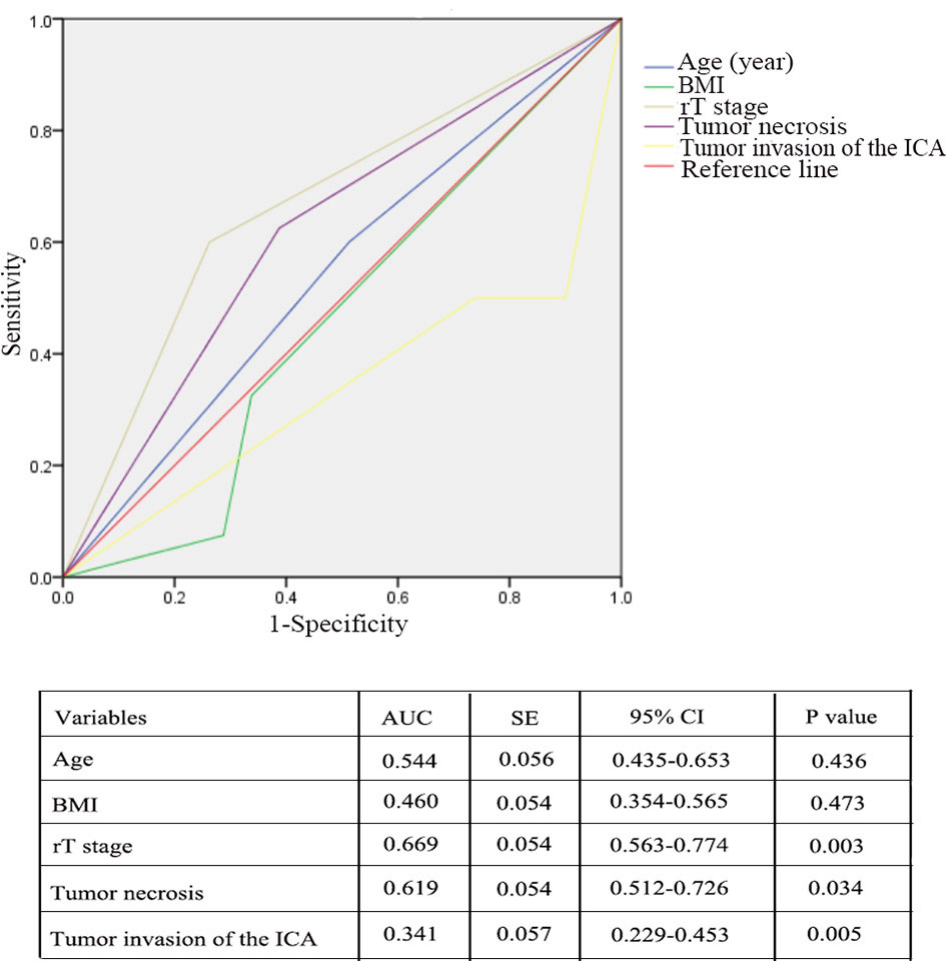
Receiver operating characteristic analysis revealed that the rT stage was the best predictor for overall survival (OS). The area under the ROC curve for rT stage was 0.669 (P = 0.003).

The variables that were considered to be significant in the Cox univariate analyses were age, BMI, rT stage, tumor necrosis, tumor invasion into the ICA, the use of pedicled flaps to repair the defect, and positive surgical margins. Subsequently, Cox multivariate analyses were performed using the aforementioned seven variables. A total of five variables (age, BMI, T stage, tumor necrosis, and tumor invasion into the ICA) were proven to be independent prognostic factors in the multivariate Cox regression model (**Table 4**). Moreover, based on the factors affecting OS, the predictive values were analyzed by means of the ROC analysis, which revealed that the rT stage was the best predictor for OS. The area under the ROC curve for rT stage with regard to the OS was 0.669 (95% confidence interval [CI], 0.563–0.774; *P*=0.003). The prognostic values pertaining to the other factors are shown in **Figure 6**. In addition, multivariable analysis revealed that patients with BMI and rT stage were significantly related to PFS (**Table 5**).

**Table 4:** Univariate and multivariate Cox regression analyses of OS in patients with rNPC.

Variable	Univeriate				Multivariate		
	HR	95%CI	P		HR	95%CI	P
Age (year)							
≥50	1		Reference		1		Reference
<50	0.517	0.272-0.985	0.045		0.462	0.235-0.908	0.025
BMI							
18.5–24.9	1		Reference		1		Reference
<18.5	3.693	1.745-7.817	0.001		2.981	1.282-6.931	0.011
>24.9	0.348	0.105-1.147	0.083		0.570	0.159-2.047	0.388
T stage							
rT3	1		Reference		1		Reference
rT4	2.827	1.483-5.391	0.002		2.396	1.140-5.037	0.021
Tumor necrosis							
Yes	1		Reference		1		Reference
No	0.361	0.189-0.688	0.002		0.488	0.249-0.960	0.038
Tumor invaded the ICA							
No invasion	1		Reference		1		Reference
Invasion	5.164	2.713-9.827	0.000		2.445	1.093-5.469	0.030
Invasion, but ICA was embolized.	<0.001	/	0.968		<0.001	/	0.971
Surgical margin							
Negative margin	1		Reference		1		Reference
Positive margin	2.108	1.123-3.959	0.020		1.017	0.478-2.162	0.965
The use of pedicled flap							
Yes	1		Reference		1		Reference
No	2.179	1.085-4.379	0.029		1.405	0.666 -2.968	0.372
Sex							
Male	1		Reference				
Female	1.667	0.876-3.171	0.119				
Smoking history							
Yes	1		Reference				
No	2.064	0.805-5.295	0.132				
Drinking history							
Yes	1		Reference				
No	2.439	0.751-7.926	0.138				
Diabetes mellitus							
Yes	1		Reference				
No	0.911	0.124-6.685	0.911				
Hypertension							
Yes	1		Reference				
NO	0.933	0.455-1.910	0.849				
Hemoglobin							
<120	1		Reference				
≥120	0.783	0.405-1.511	0.466				
NLR							
≥6	1		Reference				
<6	0.593	0.294-1.198	0.145				
Alkaline phosphatase							
<50	1		Reference				
≥50	1.096	0.264-4.552	0.900				
The time between recurrence and the last radiotherapy (year)							
≥3	1		Reference				
<3	1.282	0.684-2.405	0.438				
Preoperative combined chemotherapy before surgery							
Yes	1		Reference				
No	1.922	0.937-3.941	0.075				
Histological subtype							
WHO type II	1		Reference				
WHO type III	0766	0.405-1.447	0.411				
LNM							
Yes	1		Reference				
No	0.657	0.334-1.292	0.068				
Postoperative radiotherapy							
No	1		Reference				
Yes	1.305	0.401-4.240	0.658				
Postoperative chemotherapy							
No	1		Reference				
Yes	0.498	0.195-1.273	0.145				
Postoperative PD-1 treatment							
No	1		Reference				
Yes	0.324	0.044-2.366	0.267				

rNPC, recurrent nasopharyngeal carcinoma; OS, Overall survival; BMI, Body mass index; NLR, Neutrophil to lymphocyte ratio; LNM, lymph node metastasis; ICA, Internal carotid artery; HR, hazard ratio; CI, confidence interval.

**Table 5:** Univariate and multivariate Cox regression analyses of PFS in patients with rNPC.

Variable	Univeriate			Multivariate		
	HR	95%CI	P	HR	95%CI	P
Age (year)						
≥50	1		Reference	1		Reference
<50	0.580	0.352-0.957	0.033	0.610	0.368-1.010	0.055
BMI						
18.5–24.9	1		Reference	1		Reference
<18.5	2.795	1.445-5.409	0.002	2.462	1.267-4.784	0.008
>24.9	1.019	0.544-1.908	0.953	0.904	0.480-1.703	0.755
T stage						
rT3	1		Reference	1		Reference
rT4	1.750	1.075-2.850	0.024	1.648	1.010-2.689	0.046
Sex						
Male	1		Reference			
Female	0.881	0.511-1.518	0.648			
Smoking history						
Yes	1		Reference			
No	0.662	0.384-1.142	0.138			
Drinking history						
Yes	1		Reference			
No	0.849	0.477-1.839	0.849			
Diabetes mellitus						
Yes	1		Reference			
No	0.849	0.207-3.480	0.849			
Hypertension						
Yes	1		Reference			
No	0.677	0.496-1.578	0.677			
Hemoglobin						
<120	1		Reference			
≥120	0.670	0.401-1.119	0.126			
NLR						
≥6	1		Reference			
<6	0.872	0.482-1.579	0.652			
Alkaline phosphatase						
<50	1		Reference			
≥50	1.732	0.424-7.084	0.444			
The time between recurrence and the						
last radiotherapy (year)						
≥3	1		Reference			
<3	1.292	0.794-2.101	0.303			
Preoperative combined chemotherapy before surgery						
Yes	1		Reference			
No	1.211	0.645-2.275	0.551			
Histological subtype						
WHO type II	1		Reference			
WHO type III	1.289	0.755-2.200	0.353			
LNM						
Yes	1		Reference			
No	0.688	0.402-1.175	0.171			
Postoperative radiotherapy						
No	1		Reference			
Yes	0.559	0.223-1.402	0.215			
Postoperative chemotherapy						
No	1		Reference			
Yes	1.374	0.790-2.390	0.261			
Postoperative PD-1 treatment						
No	1		Reference			
Yes	0.839	0.361-1.947	0.683			
Tumor necrosis						
Yes	1		Reference			
No	0.910	0.558-1.483	0.705			
Tumor invaded the ICA						
No invasion	1		Reference			
Invasion	1.554	0.888-2.718	0.122			
Invasion, but ICA was embolized.	0.774	0.306-1.959	0.589			
Surgical margin						
Negative margin	1		Reference			
Positive margin	1.444	0.875-2.383	0.150			
The use of pedicled flap						
Yes	1		Reference			
No	0.853	0.523-1.391	0.853			

rNPC, recurrent nasopharyngeal carcinoma; PFS, progress free survival; BMI, Body mass index; NLR, Neutrophil to lymphocyte ratio; LNM, lymph node metastasis; ICA, Internal carotid artery; HR, hazard ratio; CI, confidence interval.

## Discussion

Aggressive salvage treatment is recommended for the management of locally rNPC, owing to the fact that many patients can still achieve long-term survival (4). The most common therapeutic modalities used for the management of rNPC are salvage surgery and re-irradiation with IMRT. Moreover, several studies have reported that IMRT is the most effective treatment for the management of advanced rNPC. Salvage surgery is only employed in the scenarios involving resectable, early-stage rNPC, such as rT1 disease, rT2–3 disease with limited parapharyngeal space involvement, or disease confined to the base of the sphenoid sinus (11, 12). However, advanced rNPC is characteristically more radioresistant, compared to the primary tumor, which can be attributed to the poor blood supply and hypoxia, and a suboptimal dose distribution resulting from the protection of critical structures. Re-irradiation for the management of these bulky tumors warrants a higher radiation dose and wider treatment range to include a 1–1.5 cm margin, thereby compromising adjacent critical structures, which leads to severe radiotherapy-related toxicity and poor survival outcomes (13–15).

Over the recent years, several studies have suggested that salvage surgery could be used in patients with advanced rNPC (9, 10, 12, 16). Wong et al. reported favorable patient outcomes in fifteen patients with advanced rNPC (two rT3 and thirteen rT4 tumors) who underwent surgery by means of the endoscopic approach; the two-year OS was 66.7% (10). In our study, the three-year OS in patients with advanced rNPC was 55.2%, which was higher, compared to the patients who underwent salvage IMRT (17–19). Furthermore, the three-year OS in patients with rT3 and rT4 tumors were 68.8% and 36.9%, respectively, which were higher, compared to the patients who underwent salvage IMRT pertaining to rT3 and rT4 tumors, i.e., of 49.5% and 33.9%, reported by Hua et al. (18) However, many patients who received salvage surgery were given postoperative multimodal treatment including chemotherapy, radiotherapy or anti-PD-1 drugs. In addition, this study lacked a comparison cohort between salvage surgery and IMRT. Thus, case matching studies and prospective studies with larger clinical samples are required to further evaluate the efficacy of endoscopic surgery compared with re-irradiation in advanced rNPC.Postoperative ICA hemorrhage is one of the major causes of mortality after salvage surgery in patients with advanced rNPC. Consequently, tumor invasion into the ICA presents a difficult undertaking with regard to surgery. During the early years of the current research, the present study adopted the vidian nerve and eustachian tube as consistent and reliable ICA markers in the patients undergoing such high-risk surgical procedures (20). According to the relationship between the aforementioned markers, the parapharyngeal segment and petrous segment can be safely identified during the surgical procedure. However, the tumor resection had limitations; the tumor close to the ICA could only be removed as much as possible. Inevitably, some patients had tumor recurrence or postoperative ICA hemorrhage, which affects the OS in patients with advanced rNPC.

Recently, the authors proposed a new surgical procedure for the management of rNPC, namely, the embolization of ICA and the resection of tumor invading the ICA. This innovative technique can expand the scope of surgical resection and completely remove the tumor on the ICA, so as to ensure the negative margin, and reduce the probability of residual tumor and recurrence. In addition, it can avoid the occurrence of ICA hemorrhage in the process of tumor dissection, thus reduce the risk of surgery. More importantly, preoperative radiotherapy and previous surgical trauma have a great influence on the blood supply to the skull base, and postoperative cavity infection can occur easily. When the focus of infection invades the ICA, it leads to fatal ICA hemorrhage. Consequently, ICA embolization can also prevent postoperative ICA rupture. In this series, the three-year OS rate pertaining to the patients without ICA invasion, invasion into ICA, and embolization of ICA were 65.1%, 15.7%, and 100%, respectively. Multivariate Cox regression analysis showed that ICA invasion was an independent risk factor for OS. Hence, it was concluded that the patients with rNPC with ICA invasion who undergo preoperative ICA embolization might have a better survival prognosis after salvage nasopharyngectomy.

BMI is a simple weight-for-height calculation that is often used to evaluate the nutritional status in adults. Several studies have reported that lower BMI is an independent risk factor for tumor recurrence or distant metastasis after radiotherapy in NPC patients (21, 22). A previous study regarding metastatic NPC by Li et al. demonstrated that the overweight/obese status was associated with longer OS, compared to the underweight or normal weight status. Overweight patients experience adverse treatment outcomes to a lesser extent and are less likely to experience malnutrition, cachexia, and increased tolerance to cancer treatment (23). The current study observed that rNPC patients with low BMI displayed worse OS after endoscopic surgery, compared to the patients with normal BMI. The patients with low BMI should receive further assessment, intensive counseling, and nutrition support. However, the current study did not observe any significant difference between the patients with high BMI and normal BMI with regard to OS, which was consistent with our preliminary report (24, 25).

## Conclusion

Generally, Salvage treatment using endoscopic nasopharyngectomy appears to be an effective treatment in the management of patients with advanced rNPC. The independent risk factors pertaining to the OS were age (above 50 years), low BMI, rT4 stage, tumor necrosis, and tumor invasion into the ICA. In addition, case matching studies and prospective studies with larger clinical samples are required to further evaluate the efficacy of endoscopic surgery compared with re-irradiation in advanced rNPC.

## Data Availability Statement

The original contributions presented in the study are included in the article/supplementary material. Further inquiries can be directed to the corresponding authors.

## Ethics Statement 

The studies involving humans were reviewed and approved by the Institutional Review Board of AEENTH at Fudan University. The patients/participants provided their written informed consent to participate in this study.

## Author Contributions

DW, HY, and XS conceived, designed, and supervised the study. WL conceived and designed the study, performed the statistical analysis, and drafted the manuscript. HZ conceived and designed the study and drafted the manuscript. HLu acquired the data. YG and HLi drafted the manuscript. All authors contributed to the article and approved the submitted version.

## Funding

This work was financially supported by the National Natural Science Foundation of China (No. 81870703), Shanghai Shen Kang Hospital Development Center (SHDC12018118), Clinical Research Plan of SHDC (SHDC2020CR2005A), Research Units of New Technologies of Endoscopic Surgery in Skull Base Tumor (2018RU003) supported by the Chinese Academy of Medical Sciences, Science and Technology Commission of Shanghai Municipality (20Y11902000, 21ZR1411700), and Shanghai Municipal Health Commission (201940143).

## Conflict of Interest

The authors declare that the research was conducted in the absence of any commercial or financial relationships that could be construed as a potential conflict of interest.
